# *N*-(4-Bromo­phen­yl)-2-[(1-cyclo­hexyl­meth­yl-1*H*-1,2,4-triazol-3-yl)sulfanyl]­acetamide

**DOI:** 10.1107/S1600536812015991

**Published:** 2012-04-21

**Authors:** Yue-Ping Wang, Wan-Lu Yan, Qiong Guo, Yan-Ping He

**Affiliations:** a Department of Applied Chemistry, Faculty of Science, Kunming University of Science and Technology, Kunming, 650050, People’s Republic of China; b Key Laboratory of Medicinal Chemistry for Natural Resource, (Ministry of Education), School of Chemical Science and Technology, Yunnan University, Kunming 650091, People’s Republic of China

## Abstract

The title compound, C_17_H_21_BrN_4_OS, was synthesized as a potential reverse transcriptase (RT) inhibitor of the human immunodeficiency virus type 1 (HIV-1). In the molecule, there is an N—H⋯S hydrogen bond making a five-membered ring. In the crystal, mol­ecules are connected into centrosymmetric dimers *via* pairs of N—H⋯N and weak C—H⋯N hydrogen bonds. The crystal structure also features C—H⋯O inter­actions.

## Related literature

The 1,2,4-triazole scaffold and its analogues are important pharmacophores that can be found in biologically active compounds across a number of different therapeutic areas, see: Lin *et al.* (2005 ▶); Naito *et al.* (1996 ▶); Sui *et al.* (1998 ▶); Tafi *et al.* (2002 ▶).
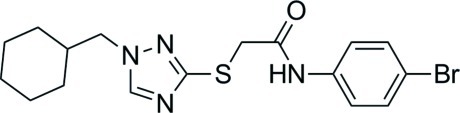


## Experimental

### 

#### Crystal data

C_17_H_21_BrN_4_OS
*M* *_r_* = 409.35
Triclinic, 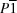

*a* = 7.2061 (8) Å
*b* = 9.521 (1) Å
*c* = 14.2862 (16) Å
α = 104.132 (1)°
β = 90.804 (1)°
γ = 95.820 (1)°
*V* = 944.84 (18) Å^3^
*Z* = 2
Mo *K*α radiation
μ = 2.30 mm^−1^
*T* = 298 K
0.25 × 0.16 × 0.12 mm

#### Data collection

Bruker SMART CCD area-detector diffractometer
Absorption correction: multi-scan (*SADABS*; Bruker, 1998 ▶) *T*_min_ = 0.597, *T*_max_ = 0.770
8793 measured reflections
4076 independent reflections
2644 reflections with *I* > 2σ(*I*)
*R*_int_ = 0.025

#### Refinement

*R*[*F* ^2^ > 2σ(*F* ^2^)] = 0.038
*wR*(*F* ^2^) = 0.092
*S* = 1.01
4076 reflections
217 parameters
H-atom parameters constrained
Δρ_max_ = 0.34 e Å^−3^
Δρ_min_ = −0.42 e Å^−3^

### 

Data collection: *SMART* (Bruker, 1998 ▶); cell refinement: *SAINT* (Bruker, 1998 ▶); data reduction: *SAINT*; program(s) used to solve structure: *SHELXS97* (Sheldrick, 2008 ▶); program(s) used to refine structure: *SHELXL97* (Sheldrick, 2008 ▶); molecular graphics: *SHELXTL* (Sheldrick, 2008 ▶); software used to prepare material for publication: *SHELXTL*.

## Supplementary Material

Crystal structure: contains datablock(s) I, global. DOI: 10.1107/S1600536812015991/bq2348sup1.cif

Structure factors: contains datablock(s) I. DOI: 10.1107/S1600536812015991/bq2348Isup2.hkl

Additional supplementary materials:  crystallographic information; 3D view; checkCIF report

## Figures and Tables

**Table 1:** Hydrogen-bond geometry (Å, °)

*D*—H⋯*A*	*D*—H	H⋯*A*	*D*⋯*A*	*D*—H⋯*A*
N4—H4⋯S1	0.86	2.61	3.096 (2)	117
N4—H4⋯N1^i^	0.86	2.55	3.339 (3)	153
C1—H1⋯O1^ii^	0.93	2.29	3.214 (3)	171
C13—H13⋯N1^i^	0.93	2.48	3.342 (3)	153

Symmetry codes: (i) 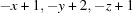; (ii) 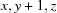.

**Fig. 1.**
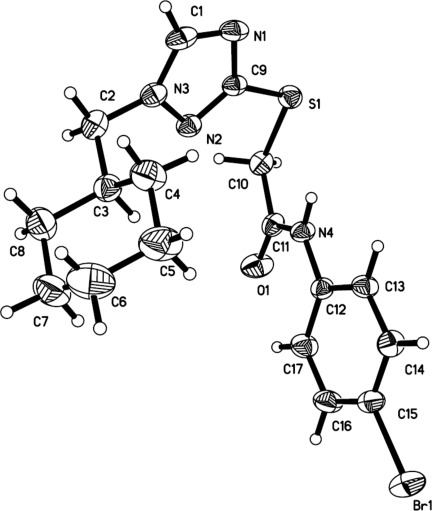
The molecular structure of the title compound, showing the atom labeling scheme and 30% probability displacement ellipsoids.

**Fig. 2.**
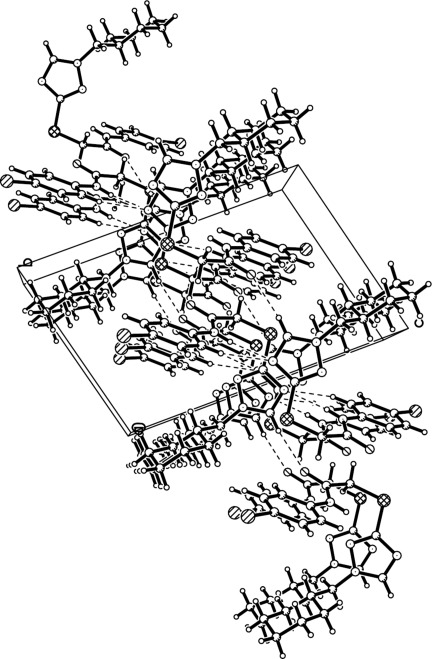
The crystal packing of the title compound, viewed along the a axis. Intermolecular N—H···Ni, C—H···Ni, C—H···Oii hydrogen bond are shown as dashed lines [symmetry codes: (i) -x + 1, -y + 2, -z + 1; (ii) x, 1 + y, z].

## supplementary crystallographic information

### Comment

The 1,2,4-triazole scaffold and its analogues are important pharmacophores that can be found in biologically active compounds across a number of different therapeutic areas; these include antifungal (Tafi, *et al.*, 2002), antibacterial (Sui, *et al.*, 1998), antiasthmatic (Naito, *et al.*, 1996) and anticancer activities (Lin, *et al.*, 2005), *etc*. In the course of our search for new anti-HIV-1 agents, we have synthesized a new series of 1,2,4-triazole analogues, including the title compound, (I), as potential HIV-1 inhibitors.

The chemical structure of (I) is shown in Fig.1. The molecule is stabilized by a weak intramolecular N4—H4···S1 hydrogen bond (Table 1). The cyclohexyl ring adopts the lowest energy chair conformation. The torsion angle (C1—N3—C2—C3), which describes the arrangement between the cyclohexyl ring and the triazole moiety, is -112.1 (3) °; the torsion angle C12—N4—C11—O1, which characterizes the location of the CONH_2_ group relative to the phenyl ring, is 3.5 (4) °.

In the crystal structure, centrosymmetric dimmers are formed by pairs of N4—H4···N1^i^ and C13—H13···N1^i^ hydrogen bonds [symmetry code: (i) -*x* + 1, -*y* + 2,-*z* + 1]. These dimmers are further linked into chains by weak C—H···O interactions (Table 1, Fig.2).

### Experimental

To a stirred solution of 2-((1*H*-1,2,4-triazol-3-yl)thio)-*N*-(4-bromophenyl)acetamide (3.12 g, 10 mmol) in anhydrous EtOH (75 ml) was added K_2_CO_3_ (1.38 g, 0.01 mol) under a nitrogen atmosphere. The mixture was stirred at 298 K for 15 min, then (bromomethyl)cyclohexane (0.53 g, 0.03 mol) was added, and the reaction mixture was refluxed for 8 h under a nitrogen atmosphere. The reaction mixture was poured into cold H_2_O (80 ml), then the aqueous phase was extracted with EtOAc (3×50 ml). The combined organic layer was washed with H_2_O (3×50 ml), dried (Mg_2_SO_4_), filtered and concentrated *in vacuo* to give the crude product, which was purified by chromatography (eluent EtOAc/PE 100:25) to afford the title compound (yield: 23%; m.p. 398.7–398.9 K). Single crystals of (I) suitable for X-ray diffraction were grown from a solution in EtOAc by slow evaporation. The product was characterized by IR, MS, ^1^H NMR and ^13^C NMR.

### Refinement

All H atoms were placed in calculated positions with N—H = 0.86 Å and C—H = 0.93–0.98 Å, and with Uiso(H) = 1.2Ueq(C, N).

### Crystal data

**Table:**

C_17_H_21_BrN_4_OS	*Z* = 2
*M**_r_* = 409.35	*F*(000) = 420
Triclinic, *P*1	*D*_x_ = 1.439 Mg m^−^^3^
Hall symbol: -P 1	Melting point: 398.8(1) K
*a* = 7.2061 (8) Å	Mo *K*α radiation, λ = 0.71073 Å
*b* = 9.521 (1) Å	Cell parameters from 2212 reflections
*c* = 14.2862 (16) Å	θ = 2.3–24.5°
α = 104.132 (1)°	µ = 2.30 mm^−^^1^
β = 90.804 (1)°	*T* = 298 K
γ = 95.820 (1)°	Block, colourless
*V* = 944.84 (18) Å^3^	0.25 × 0.16 × 0.12 mm

### Data collection

**Table:**

Bruker SMART CCD area-detector diffractometer	4076 independent reflections
Radiation source: fine-focus sealed tube	2644 reflections with *I* > 2σ(*I*)
Graphite monochromator	*R*_int_ = 0.025
phi and ω scans	θ_max_ = 27.0°, θ_min_ = 1.5°
Absorption correction: multi-scan (*SADABS*; Bruker, 1998)	*h* = −9→8
*T*_min_ = 0.597, *T*_max_ = 0.770	*k* = −12→12
8793 measured reflections	*l* = −18→18

### Refinement

**Table:**

Refinement on *F*^2^	Primary atom site location: structure-invariant direct methods
Least-squares matrix: full	Secondary atom site location: difference Fourier map
*R*[*F*^2^ > 2σ(*F*^2^)] = 0.038	Hydrogen site location: inferred from neighbouring sites
*wR*(*F*^2^) = 0.092	H-atom parameters constrained
*S* = 1.01	*w* = 1/[σ^2^(*F*_o_^2^) + (0.0382*P*)^2^ + 0.1448*P*] where *P* = (*F*_o_^2^ + 2*F*_c_^2^)/3
4076 reflections	(Δ/σ)_max_ = 0.001
217 parameters	Δρ_max_ = 0.34 e Å^−^^3^
0 restraints	Δρ_min_ = −0.42 e Å^−^^3^

### Special details

**Table:**

Geometry. All e.s.d.'s (except the e.s.d. in the dihedral angle between two l.s. planes) are estimated using the full covariance matrix. The cell e.s.d.'s are taken into account individually in the estimation of e.s.d.'s in distances, angles and torsion angles; correlations between e.s.d.'s in cell parameters are only used when they are defined by crystal symmetry. An approximate (isotropic) treatment of cell e.s.d.'s is used for estimating e.s.d.'s involving l.s. planes.
Refinement. Refinement of *F*^2^ against ALL reflections. The weighted *R*-factor *wR* and goodness of fit *S* are based on *F*^2^, conventional *R*-factors *R* are based on *F*, with *F* set to zero for negative *F*^2^. The threshold expression of *F*^2^ > σ(*F*^2^) is used only for calculating *R*-factors(gt) *etc*. and is not relevant to the choice of reflections for refinement. *R*-factors based on *F*^2^ are statistically about twice as large as those based on *F*, and *R*- factors based on ALL data will be even larger.

### Fractional atomic coordinates and isotropic or equivalent isotropic displacement parameters (Å^2^)

**Table:**

	*x*	*y*	*z*	*U*_iso_*/*U*_eq_	
Br1	1.07267 (5)	0.57798 (4)	0.86425 (2)	0.07787 (16)	
N1	0.2679 (3)	1.0737 (2)	0.49703 (17)	0.0506 (6)	
N2	0.1739 (3)	0.9382 (2)	0.60131 (15)	0.0444 (5)	
N3	0.1580 (3)	1.0832 (2)	0.64012 (16)	0.0461 (5)	
N4	0.4730 (3)	0.6839 (2)	0.59376 (15)	0.0434 (5)	
H4	0.5020	0.7572	0.5696	0.052*	
O1	0.2434 (3)	0.5032 (2)	0.59473 (15)	0.0683 (6)	
S1	0.28308 (9)	0.78377 (7)	0.42896 (5)	0.04868 (18)	
C1	0.2140 (4)	1.1586 (3)	0.5772 (2)	0.0532 (7)	
H1	0.2151	1.2590	0.5881	0.064*	
C2	0.0908 (4)	1.1312 (3)	0.7364 (2)	0.0557 (7)	
H2A	−0.0288	1.0766	0.7404	0.067*	
H2B	0.0714	1.2333	0.7483	0.067*	
C3	0.2247 (4)	1.1117 (3)	0.81422 (19)	0.0537 (7)	
H3	0.2486	1.0092	0.7988	0.064*	
C4	0.4098 (4)	1.2034 (4)	0.8179 (2)	0.0726 (9)	
H4A	0.3883	1.3049	0.8277	0.087*	
H4B	0.4685	1.1743	0.7565	0.087*	
C5	0.5408 (5)	1.1879 (5)	0.8986 (3)	0.0958 (12)	
H5A	0.5750	1.0892	0.8847	0.115*	
H5B	0.6540	1.2537	0.9016	0.115*	
C6	0.4501 (7)	1.2220 (5)	0.9949 (3)	0.1066 (14)	
H6A	0.5329	1.2047	1.0440	0.128*	
H6B	0.4294	1.3241	1.0124	0.128*	
C7	0.2675 (6)	1.1298 (5)	0.9914 (2)	0.0935 (12)	
H7A	0.2096	1.1578	1.0531	0.112*	
H7B	0.2901	1.0285	0.9808	0.112*	
C8	0.1356 (5)	1.1455 (4)	0.9123 (2)	0.0755 (9)	
H8A	0.1012	1.2442	0.9268	0.091*	
H8B	0.0227	1.0797	0.9098	0.091*	
C9	0.2392 (3)	0.9403 (3)	0.51580 (18)	0.0405 (6)	
C10	0.1917 (4)	0.6457 (3)	0.48677 (19)	0.0465 (6)	
H10A	0.0761	0.6752	0.5154	0.056*	
H10B	0.1590	0.5573	0.4363	0.056*	
C11	0.3064 (4)	0.6054 (3)	0.56361 (19)	0.0435 (6)	
C12	0.6046 (3)	0.6588 (2)	0.66041 (17)	0.0397 (6)	
C13	0.7805 (3)	0.7356 (3)	0.66796 (18)	0.0449 (6)	
H13	0.8052	0.8042	0.6320	0.054*	
C14	0.9196 (4)	0.7118 (3)	0.72794 (19)	0.0494 (6)	
H14	1.0376	0.7631	0.7319	0.059*	
C15	0.8817 (4)	0.6118 (3)	0.78159 (18)	0.0487 (6)	
C16	0.7086 (4)	0.5367 (3)	0.7767 (2)	0.0575 (7)	
H16	0.6845	0.4700	0.8141	0.069*	
C17	0.5691 (4)	0.5594 (3)	0.71644 (19)	0.0548 (7)	
H17	0.4513	0.5082	0.7134	0.066*	

### Atomic displacement parameters (Å^2^)

**Table:**

	*U*^11^	*U*^22^	*U*^33^	*U*^12^	*U*^13^	*U*^23^
Br1	0.0771 (3)	0.0900 (3)	0.0738 (2)	0.01971 (19)	−0.02021 (18)	0.03131 (19)
N1	0.0502 (13)	0.0471 (13)	0.0616 (15)	−0.0016 (11)	−0.0049 (11)	0.0299 (12)
N2	0.0453 (12)	0.0378 (12)	0.0519 (13)	0.0023 (10)	−0.0001 (10)	0.0156 (10)
N3	0.0460 (13)	0.0382 (12)	0.0539 (14)	0.0033 (10)	−0.0043 (10)	0.0119 (10)
N4	0.0402 (12)	0.0394 (11)	0.0550 (13)	0.0011 (10)	−0.0018 (10)	0.0217 (10)
O1	0.0601 (13)	0.0535 (12)	0.0978 (16)	−0.0140 (10)	−0.0139 (11)	0.0406 (12)
S1	0.0452 (4)	0.0554 (4)	0.0474 (4)	0.0041 (3)	0.0001 (3)	0.0172 (3)
C1	0.0509 (16)	0.0387 (15)	0.074 (2)	0.0001 (13)	−0.0130 (15)	0.0247 (15)
C2	0.0510 (17)	0.0502 (16)	0.0640 (19)	0.0086 (13)	0.0011 (14)	0.0091 (14)
C3	0.0556 (17)	0.0485 (16)	0.0580 (17)	0.0070 (13)	0.0034 (14)	0.0145 (13)
C4	0.060 (2)	0.087 (2)	0.074 (2)	−0.0060 (18)	−0.0076 (16)	0.0336 (18)
C5	0.070 (2)	0.128 (3)	0.098 (3)	−0.007 (2)	−0.025 (2)	0.051 (3)
C6	0.123 (4)	0.119 (3)	0.076 (3)	0.001 (3)	−0.031 (3)	0.026 (2)
C7	0.126 (4)	0.105 (3)	0.056 (2)	0.026 (3)	0.008 (2)	0.027 (2)
C8	0.083 (2)	0.078 (2)	0.066 (2)	0.0134 (19)	0.0163 (19)	0.0176 (18)
C9	0.0322 (13)	0.0427 (14)	0.0492 (15)	−0.0013 (11)	−0.0073 (11)	0.0191 (12)
C10	0.0425 (15)	0.0378 (14)	0.0566 (16)	−0.0001 (11)	−0.0033 (12)	0.0087 (12)
C11	0.0409 (15)	0.0342 (13)	0.0544 (16)	0.0046 (11)	0.0007 (12)	0.0086 (12)
C12	0.0415 (14)	0.0355 (13)	0.0446 (14)	0.0055 (11)	0.0020 (11)	0.0141 (11)
C13	0.0465 (15)	0.0410 (14)	0.0498 (15)	−0.0022 (12)	−0.0011 (12)	0.0192 (12)
C14	0.0428 (15)	0.0464 (15)	0.0581 (17)	−0.0043 (12)	−0.0051 (13)	0.0153 (13)
C15	0.0555 (17)	0.0498 (16)	0.0436 (15)	0.0113 (14)	−0.0053 (12)	0.0149 (12)
C16	0.0617 (19)	0.0613 (18)	0.0582 (18)	−0.0004 (15)	0.0003 (15)	0.0343 (15)
C17	0.0482 (16)	0.0591 (17)	0.0623 (18)	−0.0054 (14)	0.0002 (14)	0.0298 (15)

### Geometric parameters (Å, º)

**Table:**

Br1—C15	1.903 (2)	C5—H5A	0.9700
N1—C1	1.319 (3)	C5—H5B	0.9700
N1—C9	1.357 (3)	C6—C7	1.499 (5)
N2—C9	1.319 (3)	C6—H6A	0.9700
N2—N3	1.373 (3)	C6—H6B	0.9700
N3—C1	1.324 (3)	C7—C8	1.512 (5)
N3—C2	1.447 (3)	C7—H7A	0.9700
N4—C11	1.353 (3)	C7—H7B	0.9700
N4—C12	1.412 (3)	C8—H8A	0.9700
N4—H4	0.8600	C8—H8B	0.9700
O1—C11	1.215 (3)	C10—C11	1.510 (3)
S1—C9	1.750 (3)	C10—H10A	0.9700
S1—C10	1.793 (3)	C10—H10B	0.9700
C1—H1	0.9300	C12—C13	1.387 (3)
C2—C3	1.519 (4)	C12—C17	1.388 (3)
C2—H2A	0.9700	C13—C14	1.381 (3)
C2—H2B	0.9700	C13—H13	0.9300
C3—C4	1.512 (4)	C14—C15	1.371 (4)
C3—C8	1.525 (4)	C14—H14	0.9300
C3—H3	0.9800	C15—C16	1.365 (4)
C4—C5	1.525 (4)	C16—C17	1.382 (4)
C4—H4A	0.9700	C16—H16	0.9300
C4—H4B	0.9700	C17—H17	0.9300
C5—C6	1.508 (5)		
			
C1—N1—C9	101.8 (2)	C6—C7—C8	112.0 (3)
C9—N2—N3	101.66 (19)	C6—C7—H7A	109.2
C1—N3—N2	109.2 (2)	C8—C7—H7A	109.2
C1—N3—C2	130.5 (2)	C6—C7—H7B	109.2
N2—N3—C2	120.3 (2)	C8—C7—H7B	109.2
C11—N4—C12	127.3 (2)	H7A—C7—H7B	107.9
C11—N4—H4	116.4	C7—C8—C3	111.3 (3)
C12—N4—H4	116.4	C7—C8—H8A	109.4
C9—S1—C10	100.22 (12)	C3—C8—H8A	109.4
N1—C1—N3	111.7 (2)	C7—C8—H8B	109.4
N1—C1—H1	124.1	C3—C8—H8B	109.4
N3—C1—H1	124.1	H8A—C8—H8B	108.0
N3—C2—C3	112.8 (2)	N2—C9—N1	115.6 (2)
N3—C2—H2A	109.0	N2—C9—S1	123.67 (18)
C3—C2—H2A	109.0	N1—C9—S1	120.7 (2)
N3—C2—H2B	109.0	C11—C10—S1	120.69 (18)
C3—C2—H2B	109.0	C11—C10—H10A	107.2
H2A—C2—H2B	107.8	S1—C10—H10A	107.2
C4—C3—C2	112.2 (2)	C11—C10—H10B	107.2
C4—C3—C8	110.8 (3)	S1—C10—H10B	107.2
C2—C3—C8	110.2 (2)	H10A—C10—H10B	106.8
C4—C3—H3	107.8	O1—C11—N4	123.6 (2)
C2—C3—H3	107.8	O1—C11—C10	117.7 (2)
C8—C3—H3	107.8	N4—C11—C10	118.6 (2)
C3—C4—C5	111.9 (3)	C13—C12—C17	118.6 (2)
C3—C4—H4A	109.2	C13—C12—N4	117.6 (2)
C5—C4—H4A	109.2	C17—C12—N4	123.8 (2)
C3—C4—H4B	109.2	C14—C13—C12	121.1 (2)
C5—C4—H4B	109.2	C14—C13—H13	119.5
H4A—C4—H4B	107.9	C12—C13—H13	119.5
C6—C5—C4	111.2 (3)	C15—C14—C13	119.2 (2)
C6—C5—H5A	109.4	C15—C14—H14	120.4
C4—C5—H5A	109.4	C13—C14—H14	120.4
C6—C5—H5B	109.4	C16—C15—C14	120.8 (2)
C4—C5—H5B	109.4	C16—C15—Br1	119.7 (2)
H5A—C5—H5B	108.0	C14—C15—Br1	119.5 (2)
C7—C6—C5	111.2 (3)	C15—C16—C17	120.3 (2)
C7—C6—H6A	109.4	C15—C16—H16	119.9
C5—C6—H6A	109.4	C17—C16—H16	119.9
C7—C6—H6B	109.4	C16—C17—C12	120.0 (3)
C5—C6—H6B	109.4	C16—C17—H17	120.0
H6A—C6—H6B	108.0	C12—C17—H17	120.0
			
C9—N2—N3—C1	−0.3 (3)	C1—N1—C9—S1	178.04 (18)
C9—N2—N3—C2	−179.5 (2)	C10—S1—C9—N2	6.0 (2)
C9—N1—C1—N3	0.4 (3)	C10—S1—C9—N1	−172.49 (19)
N2—N3—C1—N1	−0.1 (3)	C9—S1—C10—C11	−81.3 (2)
C2—N3—C1—N1	179.1 (2)	C12—N4—C11—O1	3.5 (4)
C1—N3—C2—C3	−112.1 (3)	C12—N4—C11—C10	−176.2 (2)
N2—N3—C2—C3	67.0 (3)	S1—C10—C11—O1	−174.2 (2)
N3—C2—C3—C4	63.3 (3)	S1—C10—C11—N4	5.5 (3)
N3—C2—C3—C8	−172.7 (2)	C11—N4—C12—C13	168.5 (2)
C2—C3—C4—C5	177.8 (3)	C11—N4—C12—C17	−9.8 (4)
C8—C3—C4—C5	54.1 (4)	C17—C12—C13—C14	1.6 (4)
C3—C4—C5—C6	−54.9 (4)	N4—C12—C13—C14	−176.8 (2)
C4—C5—C6—C7	55.1 (5)	C12—C13—C14—C15	−0.8 (4)
C5—C6—C7—C8	−55.9 (5)	C13—C14—C15—C16	−0.4 (4)
C6—C7—C8—C3	55.4 (4)	C13—C14—C15—Br1	179.86 (19)
C4—C3—C8—C7	−54.1 (4)	C14—C15—C16—C17	0.7 (4)
C2—C3—C8—C7	−178.8 (3)	Br1—C15—C16—C17	−179.5 (2)
N3—N2—C9—N1	0.5 (3)	C15—C16—C17—C12	0.1 (4)
N3—N2—C9—S1	−178.05 (16)	C13—C12—C17—C16	−1.3 (4)
C1—N1—C9—N2	−0.6 (3)	N4—C12—C17—C16	177.0 (2)

### Hydrogen-bond geometry (Å, º)

**Table:**

*D*—H···*A*	*D*—H	H···*A*	*D*···*A*	*D*—H···*A*
N4—H4···S1	0.86	2.61	3.096 (2)	117
N4—H4···N1^i^	0.86	2.55	3.339 (3)	153
C1—H1···O1^ii^	0.93	2.29	3.214 (3)	171
C13—H13···N1^i^	0.93	2.48	3.342 (3)	153

Symmetry codes: (i) −*x*+1, −*y*+2, −*z*+1; (ii) *x*, *y*+1, *z*.
